# TIM-3 Promotes Proliferation of Acute Myeloid Leukemia Blasts

**DOI:** 10.3390/biomedicines13112841

**Published:** 2025-11-20

**Authors:** Zong-Yan Shi, Kai Sun, Zhao-Yu Li, Dai-Hong Xie, Ya-Zhen Qin

**Affiliations:** Peking University People’s Hospital, Peking University Institute of Hematology, National Clinical Research Center for Hematologic Disease, Beijing 100044, China

**Keywords:** acute myeloid leukemia, TIM-3, cell cycle, prognosis

## Abstract

**Background**: The immunocheckpoint TIM-3 is also expressed on acute myeloid leukemia (AML) blasts. Its prognostic significance requires clarification through subgroup analysis, while its functional roles and underlying mechanisms remain to be further investigated. **Methods:** Expression of TIM-3 was assessed in fresh bone marrow samples from 81 newly diagnosed patients with AML and 7 healthy donors using multi-color flow cytometry. TIM-3 overexpression was induced in Kasumi-1 and HL60 cell lines via lentiviral infection, and subsequent assays for cell proliferation, cell cycle, apoptosis, subcutaneous tumor formation, and Western blotting were performed. Sorted CD34^+^ cells from bone marrow mononuclear cells of 4 newly diagnosed AML patients were used for evaluating Ki67^+^ frequency with TIM-3 blocked or not. CD34^+^ cells from bone marrow mononuclear cells of other 4 newly diagnosed patients with AML were sorted into TIM-3^+^ and TIM-3^−^ cells and subjected to transcriptome sequencing. **Results:** High frequencies of CD34^+^TIM-3^+^ cells at diagnosis were related to high relapse rates in AML patients with t(8;21) (*p* = 0.025) but not in non-CBF-AML patients (*p* = 0.16). In vitro, TIM-3 upregulation in Kasumi-1 and HL60 cells enhanced cell proliferation (*p* = 0.002 and 0.013) and increased the S phase cell population (*p* = 0.006 and < 0.001), without affecting apoptosis (all *p* > 0.05). In vivo, TIM-3 upregulation promoted subcutaneous tumor formation in BALB/c nude mice, particularly in t(8;21) AML cells (*p* = 0.0068 and 0.045). In addition, blocking TIM-3 tended to decrease Ki-67^+^ frequency in CD34^+^ cells of AML patients (*p* = 0.058). KEGG enrichment analysis of transcriptome data revealed significant enrichment of cell cycle, with key genes including CDK1, CCNA2, CDCA5, AURKB, SGO1, TTK, TICRR, and NDC80 showing significantly higher expression in CD34^+^TIM-3^+^ cells compared to CD34^+^TIM-3^−^ cells. Notably, CDK1 and CCNA2, critical regulators of the cell cycle, were upregulated in TIM-3-overexpressing Kasumi-1 and HL60 cells. **Conclusions:** High TIM-3 expression in AML blasts at diagnosis is associated with relapse in the t(8;21) subtype. TIM-3 promotes AML blast proliferation by upregulating CDK1 and CCNA2, facilitating cell cycle entry.

## 1. Introduction

T-cell immunoglobulin and mucin domain-containing protein 3 (TIM-3), an immune checkpoint molecule from the TIM family, is expressed on immune cells such as T lymphocytes and natural killer cells. Additionally, its aberrantly expression across various tumor cells, including those in acute myeloid leukemia (AML), positions it as a potential therapeutic target. Being a transmembrane protein, TIM-3 exerts its effects by binding to ligands, including galectin-9 (Gal-9) and CEACAM [1].

Research on expression of TIM-3 in AML cells has shown variability in its levels across different cytogenetic subtypes, with t(8;21) and inv(16) being associated with the highest TIM-3 expression [2]. Similar findings from TCGA database analysis and flow cytometry revealed higher TIM-3 expression in CBF-AML than other subtypes [3]. However, studies in AML investigating the prognostic impact of TIM-3 have been limited by small cohort sizes and overall analysis, leading to inconsistent conclusions [3,4,5,6,7]. Given that TIM-3 expression correlated with cytogenetic profiles, evaluations should be conducted within specific subgroups.

While previous studies have explored the role of TIM-3 in solid tumors, particularly its impact on proliferation, invasion, metastasis and its mechanisms, fewer studies have focused on its role in leukemia cells. Current understanding in AML suggests TIM-3 serves as a hallmark of leukemia stem cells (LSCs), promoting LSC self-renewal through the TIM-3/Gal-9 autocrine loop [1]. However, whether TIM-3 has additional functions in AML cells, its underlying mechanisms, and its relationship to genetic background remain unresolved.

In this study, we first employed flow cytometry to assess TIM-3 expression patterns in AML blasts at diagnosis and to determine its prognostic significance in a clinical cohort. Subsequently we used in vitro and in vivo assays to investigate the impact of TIM-3 expression on the biological characteristics of AML cells as well as the underlying mechanisms.

## 2. Materials and Methods

### 2.1. Detection of TIM-3 Expression on AML Blasts by Multi-Parameter Flow Cytometry

A total of 81 non-M3 adult AML patients diagnosed between September 2020 and April 2022 were included in the study. Notably, only patients with t(8;21) were enrolled from September 2020 to November 2021, and all non-M3 patients were included from December 2021 to April 2022, resulting in an overrepresentation of RUNX1-RUNX1T1-positive patients. Diagnoses were confirmed in accordance with bone marrow morphology, immunophenotyping, cytogenetics, and molecular biology. Treatment regimens followed the standardized Chinese therapeutic guidelines [8], with follow-up extending until December 2024.

Fresh bone marrow samples from all 81 newly diagnosed AML patients as well as 7 healthy donors (HDs) were collected for TIM-3 analysis using multi-color flow cytometry (MFC). The monoclonal antibodies included CD45-V500 (BD Biosciences, Franklin Lakes, NJ, USA, Clone HI30), CD34-PerCP-Cy5.5 (Biolegend, San Diego, CA, USA, Clone 561), CD38-V450 (BD Biosciences, Clone HIT2), Lineage (Lin)-APC-H7/Cy7 with CD36 (Biolegend, Clone 5-271), CD20 (Biolegend, Clone 2H7), CD16 (BD Biosciences, Clone 3G8), CD14 (Biolegend, Clone HCD14), and CD3 (BD Biosciences, Clone SK7), IgG-APC (Biolegend, Clone M1310G05) for the control group, and TIM-3-APC (Biolegend, Clone F38-2E2) for the test group. Data collection was performed using the FACS Canto™ II (BD Biosciences, San Jose, CA, USA). Lin was used to exclude differentiated cells in the nuclear cell population (NCs), and blasts were identified as CD45dim/SSC-A-low. The frequencies of TIM-3^+^ cells were determined in Lin^−^CD34^+^ (CD34^+^), Lin^−^CD34^+^CD38^−^ (CD34^+^CD38^−^), and Lin^−^CD34^+^CD38^+^ (CD34^+^CD38^+^) cells (Figure S1).

### 2.2. Stable Transfected Cell Line Establishment and Identification

Human AML cell lines Kasumi-1 (RUNX1-RUNX1T1^+^) and HL60 (RUNX1-RUNX1T1^−^) were maintained in our laboratory. These cell lines were transfected with lentiviral particles containing TIM-3 cDNA or a control vector (GeneChem, Shanghai, China). After 12 h, the medium was replaced with fresh complete medium. Stable infected cells were selected with puromycin after 72 h of culture.

RNA from stable transfected cell lines was extracted using Trizol and reverse-transcribed to complementary DNA (cDNA). TIM-3 and control gene ABL [9] transcript levels were tested by real-time PCR using TaqMan probes, with TIM-3 transcript levels calculated using the 2^−ΔCT^ method.

### 2.3. Cell Proliferation, Cell Cycle, and Cell Apoptosis Assays

2 × 10^5^ cells were added to 6-well plates, and the fold change in cell numbers was calculated after 48 h for cell proliferation analysis.

CD34^+^ cells were sorted from the bone marrow mononuclear cells (BMMCs) of 4 newly diagnosed AML patients by flow cytometry. Then the CD34^+^ cells were incubated with or without (Con) purified anti-human TIM-3 antibody (Biolegend, San Diego, CA, USA, Clone F38.2E2) for 48 h. Ki-67-APC (Biolegend, San Diego, CA, USA, Clone Ki-67) was stained, and FACS Canto™ II (BD Biosciences, San Jose, CA, USA) was used for analysis.

After 24 h serum-free synchronized culture, 1 × 10^5^ cells were added to 6-well plates in triplicate and incubated for 72 h. Cells were then collected, washed with PBS, and incubated for 30 min in the dark with 500 μL DNA staining solution (Multisciences (Lianke) Biotech, Co., Ltd., Hangzhou, China) and 5 μL permeabilization solution (Multisciences (Lianke) Biotech, Co., Ltd., Hangzhou, China). Detection of cell cycle was performed with FACSCalibur (BD Biosciences, San Jose, CA, USA) at a flow rate of 100–300 cells/s.

Following a 48 h culture period of 1 × 10^5^ cells in 6-well plates in triplicate, cells were then collected, washed with PBS, and stained with 1 μL of Annexin V (KeyGEN BioTECH, Nanjing, China) and 2.5 μL of 7-AAD (KeyGEN BioTECH, Nanjing, China) in 100 μL binding buffer for 15 min, protected from light. After adding 200 μL binding buffer, samples were analyzed using Navios (Beckman Coulter Life Sciences, Indianapolis, IN, USA).

### 2.4. Xenograft Tumor Model

Female BALB/c nude mice, aged 4–5 weeks (Beijing Vital River Laboratory Animal Technology Co., Ltd., Beijing, China), were maintained under SPF conditions at the Peking University People’s Hospital animal facility. Prior to cell inoculation, each mouse was administered 100 mg/kg of cyclophosphamide for two consecutive days. For tumor implantation, 1 × 10^7^ cells were subcutaneously administered to the mice’s forelimb axillary areas. Control cells were seeded on the left side, and TIM-3 overexpressing cells on the right side, with three mice per group. Tumor growth was monitored daily, with tumor length and short diameter measured using Vernier calipers. Mice were euthanized when the maximum tumor diameter reached 10–20 mm. Excised tumors were weighed, volume was calculated, and photographs were taken. Then tumors were fixed, paraffin-embedded, and assessed by immunohistochemical (IHC) staining. This experiment was reviewed by the Ethics Committee of Peking University People’s Hospital, and all procedures complied with the guidelines for the use of experimental animals.

### 2.5. Flow Cytometric Sorting of CD34^+^TIM-3^+^ and CD34^+^TIM-3^−^ Cells

BMMCs were extracted from fresh bone marrow samples of four newly diagnosed adult AML patients using Ficoll-Hypaque solution (TBDsciences, Tianjin, China). Two patients were RUNX1-RUNX1T1^+^, one was CBFB-MYH11^+^, and one was FLT3-ITD^+^ NPM1^+^. All BMMCs had >1 × 10^8^ cells with high viability. To sort TIM-3^+^ and TIM-3^−^ cells in the CD34^+^ cells, BMMCs were stained with CD45-FITC (Biolegend, SanDiego, CA, USA, Clone HI30), CD34-PE-Cy7 (Biolegend, SanDiego, CA, USA, Clone 561), TIM-3-APC (Biolegend, SanDiego, CA, USA, Clone F38-2E2) for the sorting tube and IgG-APC (Biolegend, SanDiego, CA, USA, Clone M1310G05) for the control tube. Cell sorting used FACS Aria SORP (BD Biosciences, San Jose, CA, USA), with the gating strategy outlined in Figure S2. RNA was extracted from the sorted CD34^+^TIM-3^+^ and CD34^+^TIM-3^−^ cells using the RNeasy MinElute CleanUp Kit (QIAGEN, Hilden, Germany) for subsequent RNA sequencing.

### 2.6. Transcriptome Sequencing of Sorted CD34^+^TIM-3^+^ and CD34^+^TIM-3^−^ Cells

Transcriptome sequencing was carried out on eight RNA samples extracted from the sorted cells by Novogene Co., Ltd. (Beijing, China). RNA integrity and quantity were assessed using the Agilent 2100 Bioanalyzer (Santa Clara, CA, USA). PolyA-tailed mRNA was enriched using Oligo(dT) beads (Invitrogen, Thermo Fisher Scientific, Waltham, MA, USA), and cDNA was synthesized to construct sequencing libraries. The Illumina NovaSeq 6000 platform (Illumina, Inc., San Diego, CA, USA) was used to perform sequencing. After quality control of the raw sequencing data, clean reads were aligned to the hg38 reference genome using HISAT2 (version 2.0.5). Gene-level counts were generated with featureCounts (v1.5.0-p3). Fragments Per Kilobase of transcript per Million mapped reads (FPKM) values were calculated based on sequencing depth and gene length. Using DESeq2 (version 1.20.0), differential expression analysis was performed. Differentially expressed genes (DEGs) were marked as significant with a padj < 0.05 and |log2FC| ≥ 1.

### 2.7. Western Blot Assay

Proteins of cell lines were extracted by RIPA lysis buffer (Beyotime Biotechnology, Shanghai, China) with the supplement of phosphatase inhibitors, protease inhibitors, and PMSF. After quantifying protein concentration using the BCA assay, samples were subjected to immunoblotting. Primary antibodies used included anti-CDK1 (67575-1-Ig, Proteintech, Wuhan, China), anti-CCNA2 (66391-1-Ig, Proteintech, Wuhan, China), and anti-β-actin (66009-1-Ig, Proteintech, Wuhan, China). HRP-labeled goat anti-mouse (ZB-2305, ZSGB-BIO, Beijing, China) was used as the secondary antibody.

### 2.8. Statistical Analysis

Relapse-free survival (RFS) was calculated from the point of complete remission (CR) until either relapse or the final bone marrow assessment. Overall survival (OS) was measured as the time from diagnosis to death or the last follow-up. Kaplan–Meier curves and log-rank tests were used to perform survival analysis. Comparisons of continuous variables were performed with Student’s t-test and Mann–Whitney U test for two-group comparisons, and the Kruskal–Wallis test was used for multi-group comparisons. A *p*-value of <0.05 indicates statistical significance. GraphPad Prism 9 and SPSS 26.0 software were used for statistical analysis.

## 3. Results

### 3.1. TIM-3 Expression Patterns in AML Blasts

**Figure 1:**
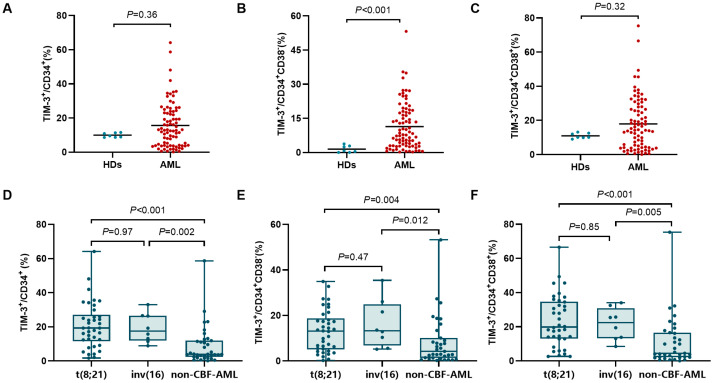
Comparison of TIM-3^+^ frequency in CD34^+^ (**A**), CD34^+^CD38^−^ (**B**), and CD34^+^CD38^+^ (**C**) cells between AML patients and HDs. Comparison of TIM-3^+^ frequency in CD34^+^ (**D**), CD34^+^CD38^−^ (**E**), and CD34^+^CD38^+^ (**F**) cells between different genetic subtypes.

The newly diagnosed AML patients showed a significantly elevated TIM-3^+^ frequency in the CD34^+^CD38^−^ population compared to HDs (median (range): 7.8% (0.21–53.2%) vs. 0.74% (0–3.8%), *p* < 0.001, Figure 1A). However, TIM-3^+^ frequencies had no significant differences in CD34^+^ (12.8% (0.18–64.2%) vs. 9.6% (8.6–11.6%), *p* = 0.36) and CD34^+^CD38^+^ (14.6% (0.10–75.4%) vs. 10.1% (9.0–13.1%), *p* = 0.32) cells between AML patients and HDs (Figure 1B,C).

Frequencies of TIM-3^+^ cells across different genetic subtypes are shown in Figure 1D–F. t(8;21) and inv(16) patients exhibited no significant difference in the frequency of CD34^+^TIM-3^+^ cells (*p* = 0.97), but both exhibited markedly higher TIM-3^+^ frequencies than non-CBF-AML patients (19.3% (1.7–64.2%) vs. 4.0% (0.18–58.7%), *p* < 0.001; 17.5% (8.9–33.1%) vs. 4.0% (0.18–58.7%), *p* = 0.002). Similarly, no significant differences in TIM-3^+^ frequencies showed in CD34^+^CD38^−^ and CD34^+^CD38^+^ cells between t(8;21) and inv(16) subtypes (*p* = 0.47 and 0.85), but both showed significantly higher TIM-3^+^ frequencies in CD34^+^CD38^−^ cells (13.1% (0.24–34.9%) vs. 4.2% (0.21–53.2%), *p* = 0.004; 13.2% (5.1–35.4%) vs. 4.2% (0.21–53.2%), *p* = 0.012) and CD34^+^CD38^+^ cells (19.9% (2.7–66.6%) vs. 4.5% (0.10–75.4%), *p* < 0.001; 22.4% (8.5–34.1%) vs. 4.5% (0.10–75.4%), *p* = 0.005) compared to non-CBF-AML.

Given the limited number of inv(16) patient samples and the similar TIM-3 expression levels in inv(16) and t(8;21) subtypes, the following prognostic analysis focused solely on t(8;21) and non-CBF-AML patients.

### 3.2. Prognostic Significance of TIM-3^+^ Frequency in AML Blasts

Out of 81 patients, 67 underwent treatment and were subsequently followed up at the institution. The diagnostic clinical characteristics of the patients were presented in Table 1. The median follow-up time was 34.3 months (range: 1.7–51.8 months). The 3-year RFS rate was 69.7% (95% CI: 55.7–80.1%), and the 3-year OS rate was 89.8% (95% CI: 78.6–95.3%).

Receiver operating characteristic (ROC) curves based on relapse were performed for all patients, t(8;21) AML patients, and non-CBF-AML patients. The optimal cutoff values for TIM-3^+^ frequencies in CD34^+^, CD34^+^CD38^−^, and CD34^+^CD38^+^ cells were determined based on the maximal Youden index (Figure S3). Patients were divided into high-frequency (-H) and low-frequency (-L) groups accordingly.

**Figure 2:**
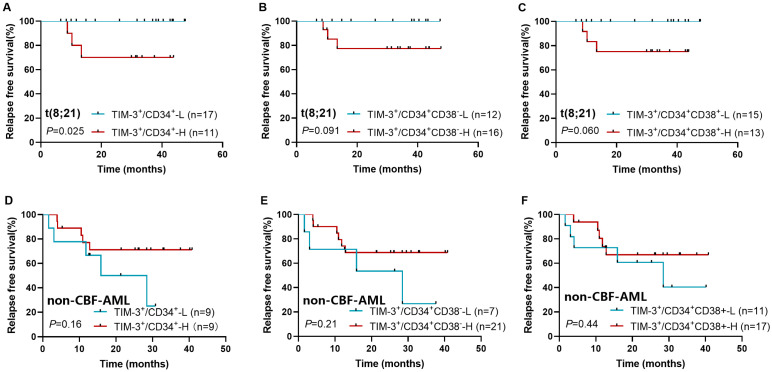
Impact of TIM-3^+^ frequency in CD34^+^ (**A**,**D**), CD34^+^CD38^−^ (**B**,**E**), and CD34^+^CD38^+^ cells (**C**,**F**) on relapse in t(8;21) AML and non-CBF-AML patients.

Among all follow-up patients, TIM-3^+^ frequencies in CD34^+^, CD34^+^CD38^−^, and CD34^+^CD38^+^ cells were not associated with RFS (*p* = 0.14, 0.13, and 0.20). In the t(8;21) subtype, a significant association was found between high CD34^+^TIM-3^+^ frequency and lower RFS (3-year RFS rate: 70.0% [95% CI: 32.9–89.2%] vs. 100% [95% CI: 100–100%], *p* = 0.025, Figure 2A). High TIM-3^+^ frequencies in CD34^+^CD38^−^ and CD34^+^CD38^+^ cells showed trends toward lower RFS (3-year RFS: 77.4% [95% CI: 44.9–92.1%] vs. 100% [95% CI: 100–100%], *p* = 0.091, Figure 2B; 75.0% [95% CI: 40.8–91.2%] vs. 100% [95% CI: 100–100%], *p* = 0.060, Figure 2C), whereas TIM-3^+^ frequency had no significant association with RFS in non-CBF-AML patients (*p* = 0.16, 0.21, and 0.44, Figure 2D–F).

### 3.3. Construction of AML Cell Lines with TIM-3 Overexpression

**Figure 3:**
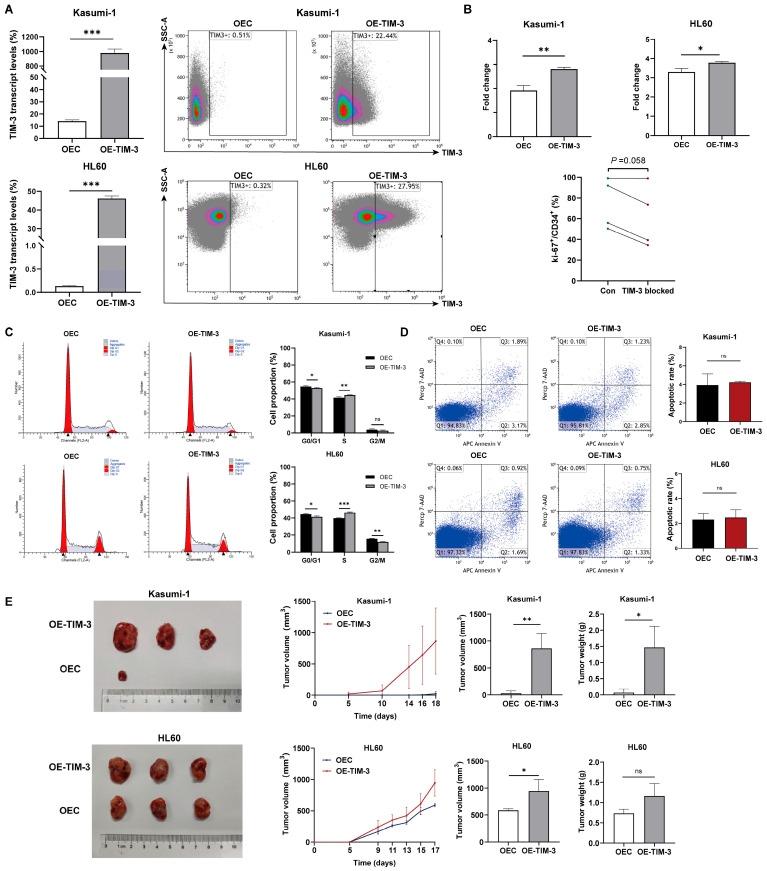
In vitro and in vivo experiments with cell lines. (**A**) TIM-3 transcript and protein levels of TIM-3 overexpressing and control cells. (**B**) Fold change in cell count after 48 h of culture in TIM-3 overexpressing and control cells, and Ki-67^+^ frequency in CD34^+^ cells of 4 AML patients with TIM-3 blocked or not. (**C**) Impact of TIM-3 overexpression on cell cycle. (**D**) Cell apoptosis in AML cell lines. (**E**) Tumor growth curves, volumes, and weights in xenograft models derived from TIM-3 overexpressing and control cells. OEC, control cells; OE-TIM-3, TIM-3 overexpressing cells. ns, no significance; * *p* < 0.05; ** *p* < 0.01; *** *p* < 0.001.

TIM-3 transcript levels in TIM-3 overexpressing cells were 69.5 times higher than in control cells in Kasumi-1 (978.7 ± 54.3% vs. 14.2 ± 1.1%, *p* < 0.001) and 348.1 times higher in HL60 (46.1 ± 1.5% vs. 0.13 ± 0.01%, *p* < 0.001) (Figure 3A). Moreover, a marked increase in TIM-3 protein expression was observed in TIM-3 overexpressing cells compared to controls in both Kasumi-1 (22.4% vs. 0.51%) and HL60 (28.0% vs. 0.32%) cells (Figure 3A). These results confirm the successful establishment of TIM-3 overexpressing Kasumi-1 and HL60 cell lines.

### 3.4. TIM-3 Promoted In Vitro and In Vivo Proliferation of AML Cells

Previous studies have shown that TIM-3 requires ligand binding to exert its function. Therefore, Gal-9 recombinant protein (MedChemExpress, USA) was included in the in vitro experiments. Preliminary results indicated that 50 pg/mL and 10 ng/mL were the optimal concentrations for Kasumi-1 and HL60 cells, respectively.

Cell viability assays revealed that TIM-3 overexpression significantly promoted cell proliferation in both Kasumi-1 (fold change: 2.8 ± 0.078 vs. 1.9 ± 0.20, *p* = 0.002) and HL60 (3.8 ± 0.066 vs. 3 ± 0.18, *p* = 0.013) cells (Figure 3B). In addition, blocking TIM-3 tended to decrease Ki-67^+^ frequency in CD34^+^ cells of AML patients (*p* = 0.058), indicating a suppression of proliferation in the primary AML blasts (Figure 3B).

Xenograft tumor models were established to assess the impact of TIM-3 expression on the tumorigenic potential of AML cells with and without the RUNX1-RUNX1T1 fusion (Figure 3E). Tumor formation in the TIM-3 overexpression Kasumi-1 group began on day 5, while only one of three control mice developed a tumor by day 18. Growth curves indicated that the TIM-3 overexpression group exhibited significantly higher tumor growth rates than the control group. Furthermore, this group showed substantially higher tumor volumes and weights compared to controls. In HL60 models, all three mice injected with either TIM-3 overexpressing or control cells developed tumors. The TIM-3 overexpression group exhibited a higher tumor growth rate, larger tumor volumes, and a tendency toward heavier tumor weights than the control group. These results demonstrate that TIM-3 overexpression enhances the tumorigenic capacity of AML cells in nude mice, with a more pronounced effect in RUNX1-RUNX1T1^+^ cells.

### 3.5. TIM-3 Promoted the Entry of AML Cells to the S Phase

The distribution of the cell cycle in Kasumi-1 and HL60 cells is shown in Figure 3C. TIM-3 upregulation significantly reduced the proportion of cells in the G0/G1 phase (52.8% ± 0.71% vs. 54.7% ± 0.85%, *p* = 0.040) and increased the proportion of cells in the S phase (55.4% ± 0.30% vs. 41.5% ± 0.96%, *p* = 0.006) in Kasumi-1 cells. Similarly, in HL60 cells, the TIM-3 overexpression group had a significantly reduced proportion of cells in the G0/G1 phase compared to the control group (41.4% ± 1.1% vs. 44.6% ± 0.65%, *p* = 0.012), while the S phase had a significantly increased cell proportion (46.5% ± 0.77% vs. 39.8% ± 0.29%, *p* < 0.001). The results indicate that TIM-3 overexpression promotes the transition of AML cells into the S phase. Furthermore, the proportion of apoptotic cells did not significantly differ between TIM-3 overexpressing cells and control cells in both Kasumi-1 and HL60 lines (Figure 3D).

### 3.6. TIM-3 Promoted Cell Cycle Progression by Up-Regulation of CDK1 and CCNA2

**Figure 4:**
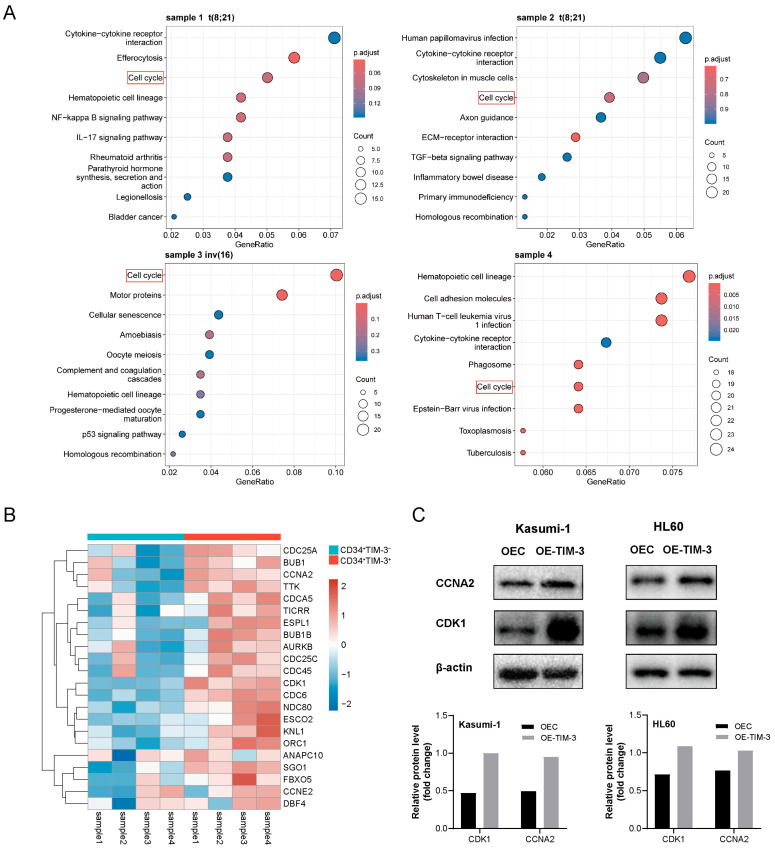
(**A**) KEGG enrichment analysis of RNA-seq data from CD34^+^TIM-3^+^ and CD34^+^TIM-3^−^ cells sorted from bone marrow samples obtained from four patients with AML. (**B**) Heatmap of differentially expressed genes associated with the cell cycle. (**C**) Western blot detection of CDK1 and CCNA2 protein in Kasumi-1 and HL60 cells. OEC, control cells; OE-TIM-3, TIM-3 overexpressing cells.

Differential gene expression analysis and enrichment analysis were carried out using RNA-seq data to identify the signaling pathways associated with TIM-3 expression in AML blasts. KEGG enrichment analysis revealed that the cell cycle pathway was significantly enriched in two t(8;21) AML samples (*p* = 0.0015 and 0.018), one inv(16) AML sample (*p* < 0.001), and one FLT3-ITD^+^ NPM1^+^ sample (*p* < 0.001) (Figure 4A). Differentially expressed genes involved in the cell cycle pathway are presented in Figure 4B, with CDK1, CCNA2, CDCA5, AURKB, SGO1, TTK, TICRR, and NDC80 significantly elevated in CD34^+^TIM-3^+^ cells compared to CD34^+^TIM-3^−^ cells. Western blot analysis confirmed the upregulation of CDK1 and CCNA2 proteins in Kasumi-1 and HL60 cells with TIM-3 overexpression (Figure 4C). IHC further demonstrated elevated CCNA2 expression in TIM-3 overexpressing cells from subcutaneous tumors both in Kasumi-1 and HL60 cells (Figure S4). These results indicated that TIM-3 promotes AML blasts to enter the cell cycle by upregulating CDK1 and CCNA2.

## 4. Discussion

AML is a clonal hematopoietic malignancy marked by significant heterogeneity. However, research into the prognostic implications of TIM-3 in AML has generally been conducted on the entire cohort, and the mechanisms of TIM-3 in AML cells are not yet clear. This study elucidated the expression patterns and clinical implications of TIM-3 in AML blasts and conducted experimental research to investigate its mechanism.

Kikushige et al. identified TIM-3 expression as specific to LSCs and not hematopoietic stem cells (HSCs), suggesting that it serves as a hallmark of LSCs [10]. In line with this, our findings demonstrated that lin^−^CD34^+^CD38^−^ cells from HDs expressed minimal TIM-3. Previous studies have also examined TIM-3 expression patterns in AML. Kikushige et al. analyzed 35 AML patients and observed high TIM-3 expression in CD34^+^CD38^−^ and CD34^+^CD38^+^ cells in nearly all M0, M1, M2, and M4 patients, whereas M5, M6, and M7 patients showed variable expression [10]. Jan et al. found that t(8;21) and inv(16) patients exhibited the highest TIM-3 transcript levels in AML [2]. Similarly, Hong et al. reported significantly higher TIM-3 levels in blasts from CBF-AML subtypes compared to non-CBF-AML based on flow cytometry and public databases [3]. Our study corroborated these findings; the TIM-3^+^ frequencies in CD34^+^, CD34^+^CD38^−^, and CD34^+^CD38^+^ cells were comparable between t(8;21) and inv(16) subtypes, with both subtypes showing significantly higher levels than non-CBF-AML patients. Given the association between TIM-3 expression and genetic subtype, subgroup analysis is essential to define its prognostic significance more accurately.

In solid tumors like hepatic cancer, gastric cancer and etc., elevated TIM-3 levels have been related to poor prognosis [11,12,13,14,15,16,17]. However, the prognostic impact of TIM-3 in AML has been assessed in a broader context with inconsistent results. Xu et al. reported that high TIM-3 levels in blasts at diagnosis correlated with high CR rates [18]. Kamal et al. found that elevated TIM-3 expression was associated with low CR rates [4], while Hong et al. observed no significant association of TIM-3 expression in AML cells with CR or OS [3]. Wu et al. identified TIM-3 as an independent predictor of poor disease-free survival (DFS) in a comprehensive analysis of the AML cohort from TCGA, and its expression in CD45^+^CD34^+^ stem/progenitor cells independently predicted event-free survival (EFS) and OS by MFC [6]. In our analysis of the entire cohort, TIM-3^+^ frequencies in CD34^+^, CD34^+^CD38^−^, and CD34^+^CD38^+^ cells were not significantly correlated with RFS. However, subtype analysis revealed that a high TIM-3^+^ frequency in these cell populations was significantly associated with higher RFS in t(8;21) patients, while no such correlation was found in non-CBF-AML patients. These findings suggest that, while expression of TIM-3 is elevated in AML blasts, it exerts a more pronounced effect in the t(8;21) subtype than in the non-CBF subtype.

To further elucidate the function of TIM-3 in different genetic subtypes of AML, both in vitro and in vivo assays were conducted. Numerous studies in solid tumors have shown that TIM-3 contributes to the proliferation, invasion, and metastasis of tumor cells [11,13,14,15,19,20,21,22,23]. Similar results have been found in hematological malignancies. Tao et al. found that HSCs in myelodysplastic syndromes exhibited high TIM-3 expression, and TIM-3^+^ HSCs exhibited abnormal differentiation, excessive proliferation, and reduced apoptosis [24]. Asayama et al. observed genes related to proliferation elevated in TIM-3^+^ cells compared to TIM-3^−^ cells in MDS blasts, and TIM-3 antibody treatment inhibited the proliferation of TIM-3^+^ blasts [25]. Additionally, silencing TIM-3 expression in HL60 cells significantly inhibited both cell proliferation and apoptosis [26]. Our results further confirmed the proliferative function of TIM-3 through both cell culture and subcutaneous tumor formation experiments, with this effect being more pronounced in the t(8;21) subtype.

To explore the underlying molecular mechanism, CD34^+^ cells from BMMC samples of four newly diagnosed AML patients were sorted into TIM-3^+^ and TIM-3^−^ populations for transcriptome sequencing. KEGG analysis revealed that the “Cell cycle” pathway was significantly enriched in all samples, suggesting a relationship between TIM-3 expression in CD34^+^ blasts and cell cycle regulation. This study further assessed the cell cycle of TIM-3 overexpressing cell lines and found that TIM-3 promotes proliferation by facilitating the transition of AML blasts into the S phase. Additionally, Western blot analysis confirmed the upregulation of CDK1 and CCNA2 proteins in TIM-3 overexpressing cells, further supporting the connection between TIM-3 expression and cell cycle regulation. The cell cycle is a four-phase process: G1, S, G2, and M Cyclin-dependent kinases (CDKs) and cyclins, which form specific complexes, play a central role in driving the cell cycle [27,28,29]. Abnormal regulation of the cell cycle is a critical mechanism underlying tumor initiation and progression. Elevated CDK activity due to DNA damage or disrupted mitotic checkpoints can drive the cell cycle in tumor cells [27]. CDK1, activated by cyclins at the end of interphase, promotes mitosis [30]. CCNA2 is a cyclin involved in initiating DNA replication and ensuring its completion during the S phase, and it also activates CDK2 during the late stages of DNA replication to promote the transition from G2 to M phase [31]. Previous studies have demonstrated the impact of TIM-3 on cyclin expression in solid tumors. Cong et al. reported that overexpression of TIM-3 in cell lines of breast cancer led to the upregulation of CCND1, which facilitated tumor cell proliferation [20]. It is hypothesized that the upregulation of CDK1 and CCNA2 in TIM-3 overexpressing AML cells contributes to the promotion of the S phase transition and the maintenance of DNA replication efficiency.

Some limitations existed in the current research. Firstly, the clinical cohort had a limited sample size, especially in t(8;21) and inv(16) subtypes. The prognostic of TIM-3^+^ frequency in AML blasts requires further confirmation by cohort with larger sample size. Secondly, the exact mechanism of TIM-3 regulation on CDK1 and CCNA2 was not explored.

In conclusion, this study highlights distinct TIM-3 expression patterns across different genetic subtypes and, for the first time, clarifies the prognostic significance of TIM-3 expression in t(8;21) AML blasts. Subsequent laboratory experiments have provided preliminary evidence that TIM-3 promotes the proliferation of AML blasts by influencing key regulatory proteins involved in the cell cycle. These findings offer new strategies and targets for enhancing AML treatment efficacy and improving prognosis.

## Figures and Tables

**Table 1:** Characteristics of patients at diagnosis and their treatment (n = 81).

Variables	Number of Patients or Median (Range)
Age (median, range, years)	40 (17–63)
Males (%)	46 (56.8%)
WBC count (median, range, ×10^9^/L)	13.4 (0.85–384.7)
Hemoglobin (median, range, g/L)	82.0 (28.0–144.0)
Platelet count (median, range, ×10^9^/L)	49.5 (4.0–507.0)
FAB types	
M2	63 (77.8%)
M4	16 (19.8%)
M5	2 (2.5%)
Genetic subtypes	
t (8;21)/RUNX1-RUNX1T1 (%)	38 (46.9%)
inv (16)/CBFB-MYH11 (%)	8 (9.9%)
MLL rearrangement (%)	4 (4.9%)
FLT3-ITD mutation (%)	23 (28.4%)
NPM1 mutation (%)	15 (18.5%)
2022 ELN genetic risk stratification * (n = 58)	
Favorable	41 (70.7%)
Intermediate	9 (15.5%)
Adverse	8 (13.8%)
CR (%)	
1-course CR	49 (77.8%)
2-course CR	11 (17.5%)
3-course CR	3 (4.7%)
Consolidation therapy	
chemotherapy	32
Allo-HSCT	31

* 23 patients were not stratified due to inadequate genetic data.

## Data Availability

The data generated during this study are available from the corresponding author upon reasonable request.

## Supplementary Materials

The following supporting information can be downloaded at: https://www.mdpi.com/article/10.3390/biomedicines13112841/s1.
