# Supplementary material for: TIM-3 Promotes Proliferation of Acute Myeloid Leukemia Blasts
Source: Biomedicines. 2025 Nov 20;13(11):2841. doi: 10.3390/biomedicines13112841 (PMC12650552; doi:10.3390/biomedicines13112841)
Supplement: Supplementary file 1 [file biomedicines-13-02841-s001.zip › biomedicines-3936891-supplementary.pdf]

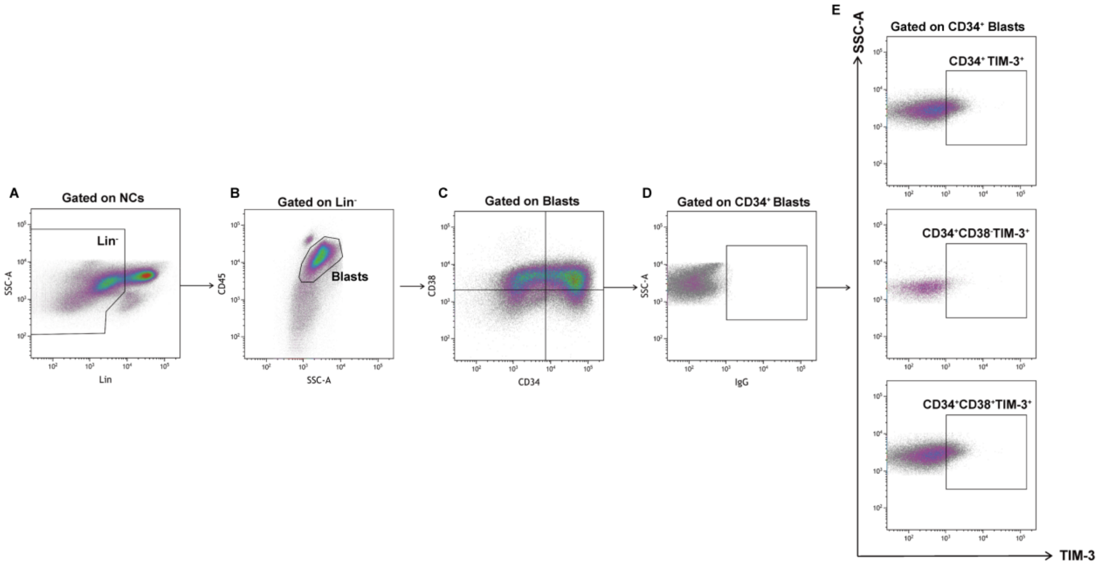

Figure S1 The gating strategy of TIM-3$^+$ frequency in CD34$^+$, CD34$^+$CD38$^-$ and CD34$^+$CD38$^+$ blasts.

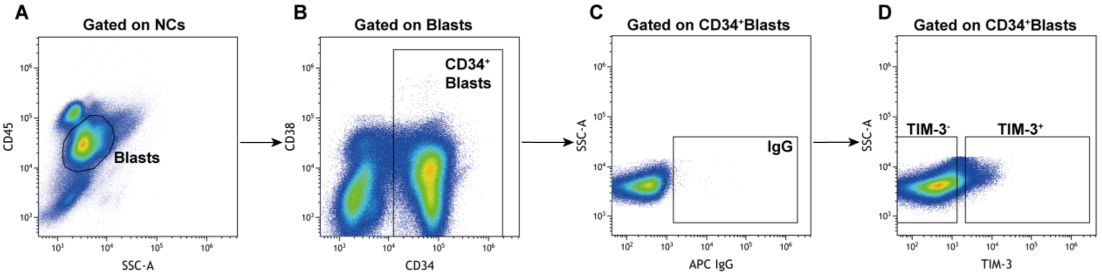

Figure S2 The gating strategy of sorting CD34$^+$TIM-3$^+$ and CD34$^+$TIM-3$^-$ cells in BMMCs samples from 4 AML patients.

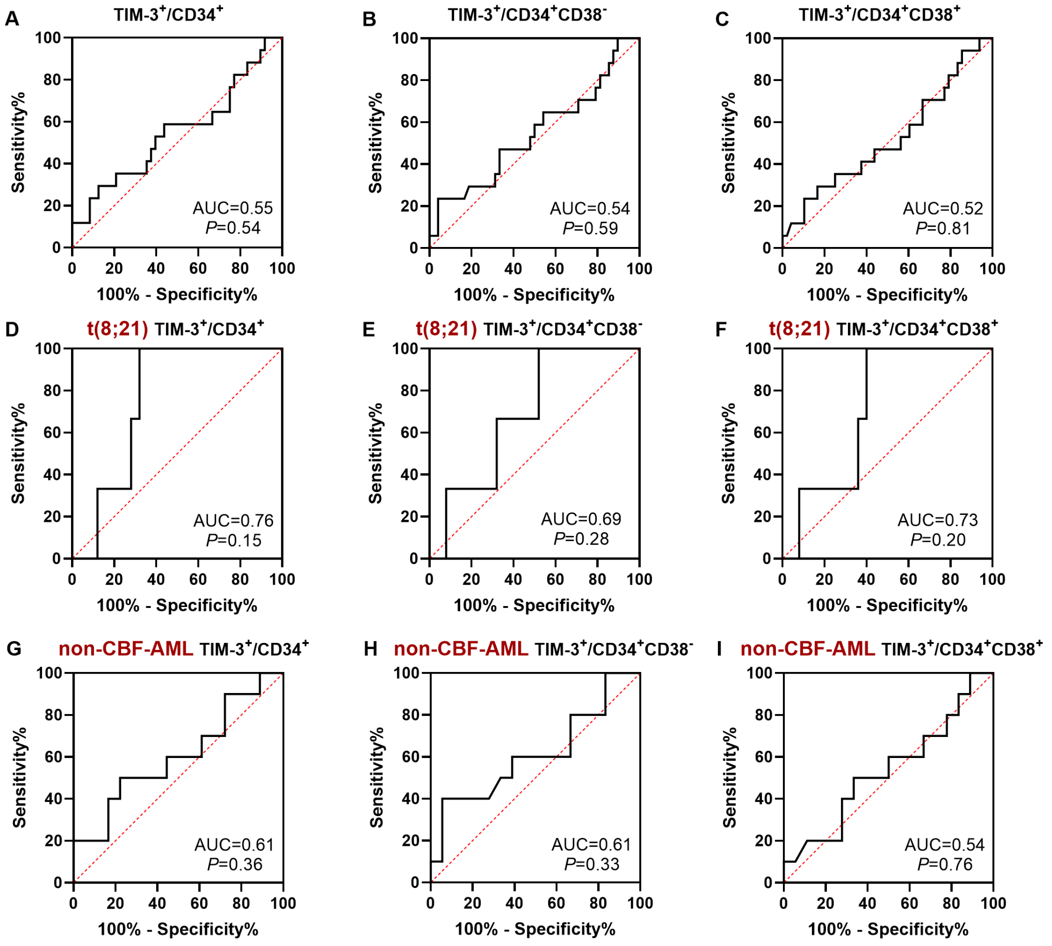

Figure S3 ROC curves of TIM-3^{+} frequency in CD34^{+}, CD34^{+}CD38^{-} and CD34^{+}CD38^{+} cells based on relapse in the whole cohort (A-C), t(8;21) patients (D-F) and non-CBF-AML patients (G-I).

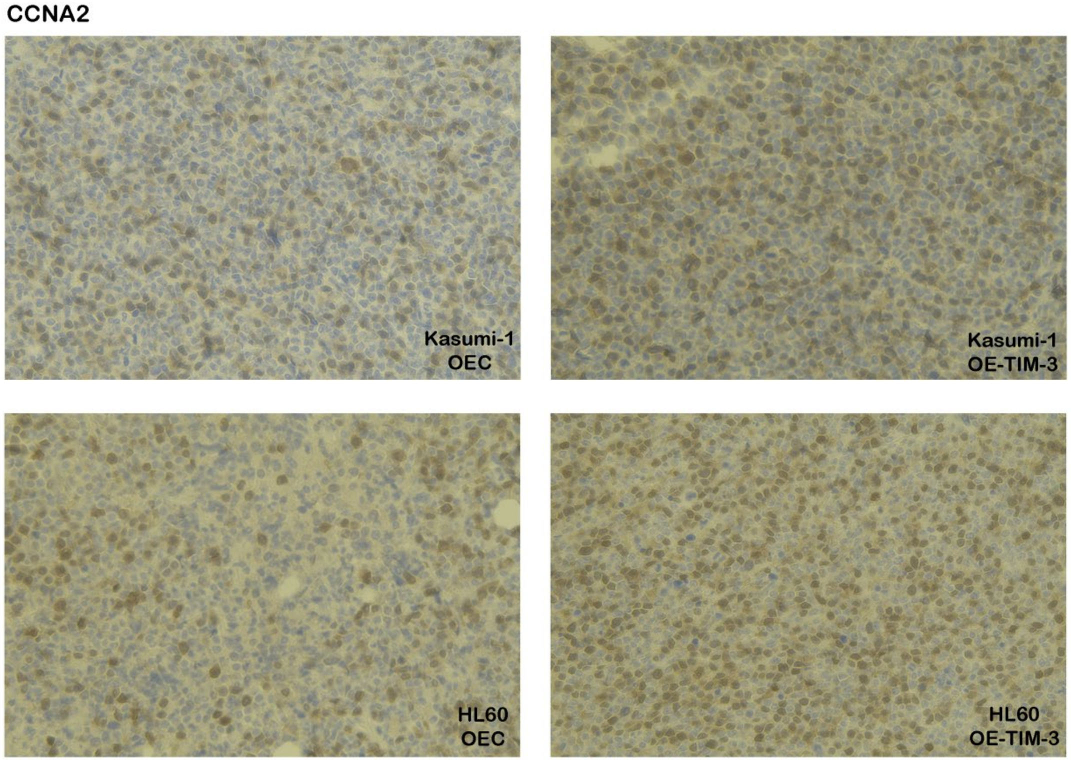

Figure S4 Immunohistochemistry staining by anti-CCNA2 antibody in TIM-3 overexpressing and control cells of subcutaneous tumors derived from Kasumi-1 and HL60 cell lines.
